# The Relationship Between Job Stress, Personality Traits and the Emotional Intelligence of Midwives Working in Health Centers of Lorestan University of Medical Sciences in 2017

**DOI:** 10.25122/jml-2018-0022

**Published:** 2018

**Authors:** Masoomeh Kheirkhah, Farzaneh Shayegan, Hamid Haghani, Ezzat Jafar Jalal

**Affiliations:** 1. Department of Nursing and Midwifery, International campus, Iran University of Medical Sciences, Tehran, Iran; 2. Department of Midwifery and Reproductive Health, Nursing Care Research Center (NCRC), Nursing and Midwifery School, Iran University of Medical Sciences, Tehran, Iran; 3. Department of Biostatistics, Iran University of Medical Sciences, Tehran, Iran; 4. Department of Nursing Management, Nursing Care Research Center (NCRC), Nursing and Midwifery School, Iran University of Medical Sciences, Tehran, Iran

**Keywords:** Job stress, emotional intelligence, and personality traits, midwife

## Abstract

**Introduction and Objective:** Job stress is one of the major threats to health and it is associated with many diseases and family problems. Midwives experience high job stress due to the management of delivery emergencies. Personality traits and emotional intelligence can be helpful in confronting environmental pressures and selecting the strategy of coping with useful stress. Thus, the current research was conducted to evaluate the relationship between job stress, personality traits and emotional intelligence in midwives of Lorestan health care centers.

**Methodology:** This research was a cross-sectional and correlational study. Midwives working in all cities of the Lorestan province were randomly selected and they completed the job stress, emotional intelligence and personality traits questionnaires. Data were analyzed using SPSS 16 software and a significance level of p <0.05 was considered.

**Results:** The results of the research showed that mean and standard deviation of job stress, emotional intelligence and personality trait midwives were 94.36 ± 12.98, 11.87 ± 14.30, and 135.51 ± 15.73 respectively. The results showed a negative relationship between intelligence and job stress (r= –0/274, p=0.0001) and no significant relationship was found between personality traits and job stress (r = –0.288, p=0.079).

**Conclusion:** A negative correlation was found between emotional intelligence and job stress, so emotional intelligence can reduce midwives’ workplace stress.

## Introduction

Despite an increase in life expectancy in communities, the average working years have decreased significantly [1]. Among the many causes, psychological factors, especially job stress, are some of the major causes of absenteeism, early retirement, and reduced working years of health workers [2]. Enhancing mental health in the work environment has been considered one of the most important dimensions of human resource development in recent decades (3). The midwifery community plays a vital role in providing the health for women and infants; it is the most important and first factor in family health and one of the components of the health team in providing services [4–5].

Due to the nature of the work, the type of tasks and responsibilities, midwives face many stressful factors each day [6–7], which might lead to a reduced quality of care provided for patients, high turnover, conflict among colleagues, health disorders, reduced correct and timely decision-making, reduced creativity and skill, job dissatisfaction, depression, feeling of inadequacy, reduced job values, fatigue, absenteeism and delay in work, increased error, reduced quality and quantity of treatment, reduced job commitment, and reduced job satisfaction [8]. Paying attention to personality traits, the way of establishing interaction with clients and the way of coping with stressful job aspects are among the factors that can be important given the job characteristics in the health system staff [9]. People’s behavior depends on their personality traits. The personality traits of staff in an organization might be a source of creativity and development or the source of conflict, failure, and inevitable organizational problems [10]. In addition, another factor which could be effective in selecting strategies to cope with stress is emotional intelligence. Emotional intelligence increases the ability and skill of people to cope with environmental pressures, and this ability to understand and manage feelings can be effective in behavior and making decisions [11].

Paying attention to the health and welfare of women, of which half the population in the community consists, is one of the most important tasks of health care managers and planners, and providing midwifery care has a direct effect on the health of mothers and infants and the community [12], and as no study has been conducted so far on the relationship between personality traits, emotional intelligence, and job stress of midwives, the present research was conducted to evaluate this relationship in midwives of Lorestan University of Medical Sciences in 2017.

## Methodology

This descriptive cross-sectional research was conducted after obtaining a license from the Ethics Committee of Iran University of Medical Sciences and an introduction letter from the health centers of Lorestan University of Medical Sciences. The multistage cluster sampling was used and the cities were selected randomly among 9 cities of the Lorestan province as a cluster, then, the health centers of these cities were selected using simple random sampling. In the next step, 200 midwives working in these centers meeting the inclusion criteria of this research were selected. The inclusion criteria of the research included having at least 6 months experience of working in health centers, having the education level of at least midwifery diploma, lack of mental problems. Absence of mental disorders for self-expression, lack of major family conflict or the death of close relatives or divorce during the last three months. The questionnaires were distributed among people who were willing to participate in the research. The research tools included a Demographic Characteristics Questionnaire, a Brad berry & Greaves Emotional Intelligence Questionnaire, a NEO-five factor inventory, a Short Form Personality Characteristics Questionnaire (NEO-FFI), and a Job Stress Questionnaire of Britain’s Health and Safety Executive (HSE). Demographic characteristics included age, marital status, education level, work experience, management history, and type of employment. Brad berry & Greaves Emotional Intelligence Questionnaires (2005) include 28 questions with four components of self-awareness (questions 1–6), self-management (questions 7–15), social awareness (questions 16–20), and relationship management (questions 21–28), scored based on 6-point Likert scale. The total scores that each subject obtained in each of the questions formed the total score of the test. A score in the range of 100 to 140 was considered excellent, a score in the range of 90 to 99 was considered good, a score in the range of 80 to 89 was considered acceptable, a score in the range of 60 to 70 requires work and effort, and a score in the range of 0 to 59 was considered a warning, which requires more attention. The validity of the tool was examined using face and content validity. In the current research, Cronbach’s alpha of the whole tool was 0.778. Scores of 0.80, 0.779, 0.776, and 0.739 were also obtained in the dimensions of self-awareness, self-management, social awareness, relationship management, respectively. The test-retest correlation coefficient was also reported as 99%. A short form of McCrae and Costa’s (1985) neo personality traits questionnaire was used, with 60 questions. Five factors included neuroticism personality, extroversion, openness to experience, agreeableness, accountability and conscientiousness and each dimension with 12 questions was scored on a five-point Likert scale (“strongly disagree” with a score of zero to “strongly agree” with a score of 4). The scores for sub-scales varied from 12–24, 24–48, and 48–60. The tool reliability of the re-test method in Garousi’s study (2006) Showed that the internal consistency in the dimensions neuroticism, agreeableness, openness, neuroticism, agreeableness to experience of the alphabet of Kronabah was 0.83, 0.75, 0.80, and 0.79 and 0.99 respectively [13]. The 99% reliability of this questionnaire was determined by the researchers through a test-retest method. Its internal consistency with Cronbach’s alpha was 74% for the whole tool and also for neuroticism, extraversion, and openness to experience, agreeableness, and accountability, respectively.

The job stress questionnaire of the UK Health and Safety Executive (HSE) includes 35 questions with 7 domains, whose dimensions of demand and expectation included topics such as workload, and work environment and characteristics with 8 items, inability to control the behaviour with 6 items, authority support with 5 items, colleagues’ support with 4 items, communication and increasing practice and positive characteristic to increase collective communication and reduce conflict in the workplace with 4 items, the individual role and correct understanding of the role of the organization’s personnel with 5 items, the way of organizing and changing the forces with 3 items were examined. The mean scores in each subscale reflect the measured value of that sub-scale, which received a score between 1 and 5 on a 5-point Likert scale. Questions (34, 22, 21, 20, 16, 14, 12, 9, 6, 5, and 3) were scored reversely. A high score reflects high job stress, and a low score reflects low stress. The reliability of the tool with Cronbach’s alpha coefficient was obtained 0.85, 0.87, 0.75, 0.63, 0.73, 0.92 in the mentioned dimensions, respectively, which confirmed Mehdizadeh et al.’s research (2013) [14]. In the current study, the tool validity was confirmed using face and content validity and its reliability by using the test re-test method was confirmed by a score of 99%. Cronbach’s alpha obtained for the total tool was 0.831, and it was 0.679, 0.677, 0.854, 0.799, 0.685, and 0.613 for the dimensions of demand and expectations, inability to control behavior, authorities support, colleagues support, communication, an individual role and understanding of the work role, changes and the way of organizing the forces, respectively. Data were analyzed using SPSS 16 software through descriptive statistics (mean and standard deviation) and correlation coefficient tests (Pearson, independent t-test and ANOVA

## Results

In this research, 44.5% of the samples were in the age group of 31–40 years old with a mean and standard deviation of 33.12 ± 7.0, of which 66% were married and 75% had the bachelor level of education. In addition, 42% of them were official employment staff, 22.5% were corporate employment staff, 14.5% were project employment staff, 12.5% of them were contract employment staff, 36.5% of them had a work experience of fewer than 5 years, 20.5% had a management history, and 12% were managers at the time of the research. Moreover, 71.5% reported an inadequate level of income and 70.5% did not receive education on job stress. The mean score of job stress in midwives was 94.36 ± 12.98, and the highest job stress among the midwives working in Lorestan University of Medical Sciences was reported in the dimension of demand and expectations, followed by communication, support of authorities, inability to control behavior, changes or the way of organizing, colleagues’ support, and the role of an individual in the organization, respectively. Based on the tool’s guideline, the score range of the tool is 35–175, and the high score reflects high stress and the low score reflects low job stress. The mean job stress among midwives is higher than the mean score of the tool (87.5), so it can be interpreted that the job stress of midwives is relatively high (Table 1).

**Table 1:** The Mean±SD of job stress among midwives working in health centers of Lorestan University of Medical Sciences in 2017

Job stress and its dimensions	min	max	Mean	SD
Demand and expectations 8–40	2.25	4/88	3/33	0/42
Inability to control behavior 6–30	1/33	4/83	2/86	0/73
Support of authorities 5–25	1	4/60	2/78	0/75
Support of colleagues 4–20	1	4/25	2/36	0/73
Communications 4–20	1	5	2/88	0/83
Individual role 5–25	1	3/60	1/62	0/62
Changes or way of organizing 3–15	1	4	2/55	0/73
Job stress 35–175	63	123	94/36	12/98

The personality trait score increased in the dimensions of neuroticism, agreeableness, openness to experience, conscientiousness, extroversion, and the highest score belonged to the extroversion or introversion dimension with a mean and standard deviation of 30.10 ± 4.29, followed by conscientiousness with a mean and standard deviation of 29.83 ± 3.58. The lowest score belonged to neuroticism with a mean and standard deviation of 21.13 ± 5.45. The mean score and standard deviation of the total personality trait were 135.51 ± 15.73, and the dominant personality traits of the midwives were extroversion and conscientiousness.

Moreover, the lowest score of emotional intelligence was seen in the social awareness dimension (20.88 ± 3.14) and the highest score was seen in the social skills dimension (35.68 ± 5.85). The mean of emotional intelligence and its standard deviation was 11.87 ± 14.30 and the emotional intelligence score increased respectively in the dimensions of social awareness, self-awareness, self-management, and social skill. Based on the tool interpretation guideline, a score higher than 100 indicates an excellent ability and a score between 90 and 99 indicates a good level of emotional intelligence. Therefore, it can be stated that midwives who participated in the study have a good to excellent emotional intelligence capability.

**Table 2:** The mean score of personality traits among midwives working in health centers of Lorestan University of Medical Sciences in 2017

Personality traits	Min	Max	Mean	SD
Neuroticism 0–48	7	45	21/13	5/45
Extroversion 0–48	20	44	30/10	4/29
Openness to experience 0–48	16	44	27/95	4/12
Agreeableness 0–48	14	47	26/64	4/85
Conscientiousness 0–48	16	44	29/83	3/58
Personality trait 0–240	97	218	135/51	15/73

## Discussion and conclusion

Severe stresses, depending on the type of personality pattern, can either interfere with the behavioral patterns and reduce self-awareness or protect people against these problems. As midwives play an important role in family and community health, by increasing the job satisfaction of midwives, the quality of care and satisfaction of pregnant mothers can be increased [15]. Of the limitations of this study, many midwives worked in health centers. After expressing the goals of repeated studying and giving the time to answer the questions by the researcher, this restriction was eliminated as far as possible.

**Table 3:** The mean score of emotional intelligence among midwives working in health centers of Lorestan University of Medical Sciences in 2017

Emotional intelligence	Min	Max	Mean	SD
Self-awareness 6–36	19	36	28/25	3/4±0/66
Self-control or self-management 9–54	23	54	33/95	3/77±0/6
Social awareness 5–30	9	28	20/88	4/18±0/62
Relation management or social skill 8–48	23	48	35/68	4/46±0/73
Emotional intelligence	86	154	111/87	4±0/51

In this research, the mean and standard deviation of job stress of midwives working in health centers of Lorestan University of Medical Sciences was 94.36 ± 12.98. In the study conducted by Golshiri et al. (2013), the job stress of nurses working in intensive care units and other female staff of Alzahra hospital in Isfahan was examined. The mean of job stress of nurses was 97.30 ± 9.29, and the mean of job stress among other female staff working in the hospital was 91.85 ± 10.91 [16]. Job stress among midwives working in health centers of the Lorestan province and the job stress among the staff of intensive care units and other staff of the Isfahan University of Medical Sciences Hospital units confirm the high level of job stress among health system staff, indicating the significance of paying attention to this group to meet the needs of the community well. The research results also showed that the mean and standard deviation of emotional intelligence was 118.77 ± 14.30, which, according to the tool interpretation guideline, can be interpreted as a good to excellent ability in emotional intelligence displayed by midwives who participated in the study.

A negative relationship between emotional intelligence and job stress was also found and with increasing emotional intelligence, job stress is reduced. This means that midwives with a higher emotional intelligence score experience less stress under the effect of changes in their organization.

Shahabi et al. (2013) conducted a correlational study on 202 faculty members of Isfahan University of Medical Sciences to determine the relationship between emotional intelligence and job stress. The results showed an inverse relationship between the score of emotional intelligence and job stress and people with high emotional intelligence experienced less job stress [17]. The dimensions of self-awareness and self-control, self-motivation, empathy, and social skills are among the factors affecting job stress. It is necessary that professional authorities take steps to strengthen this aspect through educational programs and develop plans to empower individuals in these areas. People with high emotional intelligence resist obstacles and problems. Leadership and guiding and the directing power of these people are high, and they have more willingness to participate in group activities [18].

**Table 4:** The Relationship between job stress and emotional intelligence among midwives in health centers in Lorestan University of Medical Sciences

Personality trait Job stress	Neuroticism	Extroversion/	Openness to experience	Agreebelness	Conscientiousness	Personality trait
Demand and expectations	r=0/301	r=0/149	r=0/146	r=0/326	r=0/067	r=0/300
	p=0/0001	p=0/037	p=0/041	p=0/0001	p=0/358	p=0/0001
Inability to control the behavior	r=0/013	r=–0/187	r=–0/082	r=–0/094	r=–0/256	r=–0/170
	p=0/855	p=0/009	p=0/256	p=0/190	p=0/0001	p=0/019
Support of authorities	r=0/098	r=–0/033	r=0/029	r=–0/049	r=–0/132	r=0/002
	p=0/171	p=0/649	p=0/687	p=0/492	p=0/068	p=0/979
Support of authorities	r=0/112	r=–0/044	r=0/029	r=0/032	r=–0/189	r=0/012
	p=0/117	p=0/540	p=0/686	p=0/653	p=0/009	p=0/868
Communications	r=0/329	r=0/087	r=0/219	r=0/278	r=0/068	r=0/293
	p=0/0001	P=0/223	p=0/002	p=0/0001	p=0/350	p=0/0001
Individual role in organization	r=0/218	r=–0/116	r=–0/056	r=0/017	r=–0/114	r=0/015
	p=0/002	p=0/104	p=0/432	p=0/816	p=0/116	p=0/832
The way of changing or organizing the forces	r=0/055	r=–0/207	r=–0/075	r=–0/031	r=–0/207	r=–0/108
	p=0/444	p=0/004	p=0/299	p=0/666	p=0/004	p=0/138
Job stress	r=0/245	r=–0/097	r=0/081	r=0/106	r=–0/178	r=0/079
	p=0/001	p=0/183	p=0/268	p=0/143	p=0/015	p=0/288

**Table 5:** Table 5: The Relationship between job stress and personality traits among midwives in health centers in Lorestan University of Medical Sciences

Personality trait Job stress	Neuroticism /	Extroversion	Openness to experience	Agreebelness	Conscientiousness	Personality trait
Demand and expectations	r=0/301	r=0/149	r=0/146	r=0/326	r=0/067	r=0/300
	p=0/0001	p=0/037	p=0/041	p=0/0001	p=0/358	p=0/0001
Inability to control the behavior	r=0/013	r=–0/187	r=–0/082	r=–0/094	r=–0/256	r=–0/170
	p=0/855	p=0/009	p=0/256	p=0/190	p=0/0001	p=0/019
Support of authorities	r=0/098	r=–0/033	r=0/029	r=–0/049	r=–0/132	r=0/002
	p=0/171	p=0/649	p=0/687	p=0/492	p=0/068	p=0/979
Support of authorities	r=0/112	r=–0/044	r=0/029	r=0/032	r=–0/189	r=0/012
	p=0/117	p=0/540	p=0/686	p=0/653	p=0/009	p=0/868
Communications	r=0/329	r=0/087	r=0/219	r=0/278	r=0/068	r=0/293
	p=0/0001	P=0/223	p=0/002	p=0/0001	p=0/350	p=0/0001
Individual role in organization	r=0/218	r=–0/116	r=–0/056	r=0/017	r=–0/114	r=0/015
	p=0/002	p=0/104	p=0/432	p=0/816	p=0/116	p=0/832
The way of changing or organizing the forces	r=0/055	r=–0/207	r=–0/075	r=–0/031	r=–0/207	r=–0/108
	p=0/444	p=0/004	p=0/299	p=0/666	p=0/004	p=0/138
Job stress	r=0/245	r=–0/097	r=0/081	r=0/106	r=–0/178	r=0/079
	p=0/001	p=0/183	p=0/268	p=0/143	p=0/015	p=0/288

They have high self-confidence and self-esteem and are capable of establishing beneficial relationships [19]. They can control impulsive feelings and turbulent emotions well and behave calmly. They are reliable and understand the feelings and views of others, and they can quickly change. They can adapt their responses based on the situation, are flexible and stand against the unethical acts of others. They fulfill their obligations and are responsible for the considered goals. They are honest and adapt themselves to new ideas, strategies, and information. The empathy and social skill dimension of emotional intelligence is a good predictor of job satisfaction [20]. Considering the good emotional intelligence of midwives, one can expect their optimal performance in the health system. Based on the research results, the dominant aspects of the personality trait of midwives of Lorestan Medical School were extravagance and consciousness. In the study conducted by Anisi [21], there was no significant relationship between personality traits and job stress while Ebstrup et al. had different results, finding the strongest stress-association for neuroticism [22].

The study conducted Subburaj et al. (2010) also revealed a direct and significant correlation between job stress and all five personality factors [23]. Neuroticism has a positive correlation with stress and neurotic people are more prone to job stress. In the study conducted by Jahan Bakhsh et al., a relationship was found between job stress and neuroticism [24]. On the other hand, there is a negative and significant relationship between consciousness and job stress. People with high consciousness spend much time doing their jobs, have more individual success, and less job stress [25], which is consistent with the results of current research.

## Acknowledgment

We thank the Vice-Chancellor for Research and International Affairs of Iran University of Medical Sciences and Lorestan University of Medical Sciences and all the midwives of Lorestan University of Medical Sciences whose cooperation allowed this study to happen.

## Conflict of Interest

The authors confirm that there are no conflicts of interest.

## References

[1] lmarinen J (2001). Aging workers. *Occup Environ Med*.

[2] Sjogren-Ronka T, Ojanen MT, Leskinen EK, Tmustalampi S, Malkia EA (2002). Physical andpsychosocial prerequisites of functioning in relation to work ability and general subjective well-being among office workers. *Scand J Work Environ Health*.

[3] Guo J, Chen J, Fu J, Ge X, Chen M, Liu Y (2016). Structural empowerment, job stress and burnout of nurses in China. *Applied Nursing Research*.

[4] Nourani Saadedin Sh, Hadizadeh Talasaz Z, Shakeri M., Modares Gharavi M (2013). Investigating the relationship between job stress and happiness among midwives working in hospitals and health centers in Mashhad, Iran. *Iranian Journal of Obstetrics, Genecology, and Infertility*.

[5] Hashemi Nejad N, Rahimi Moghadam S, Mohammadian M, Amiri F (2011). Investigating the relationship between mental health and job stress among midwives working in Kerman hospitals. *Iranian Journal of Obstetrics and Gynecology, and Infertility*.

[6] Chang E, Esther M, Johndaly (2005). Role stress in nurses, review of related factors and strategies for moving forward. *Journal of Nursing & Health Science*.

[7] Marit S (2008). Work distress and ethical dilemmas in neuroscience nursing. *Journal of Neuroscience Nursing*.

[8] Knezevic B, Milosevic M, Golubic R, Belosevic L, Russo A, Mustajbegovic J (2011). Work-related stress and work ability among Croatian university hospital midwives. *Midwifery*.

[9] Ulrichová M (2014). The Connection between Personality Traits and Resistance to Stress. *Procedia-Social and Behavioral Sciences*.

[10] Bosworth H B, Feaganes J R, Vitaliano P.P, Mark D.B, Siegler I.C (2001). Personality and Coping with a Common Stressor: Cardiac Catheterization. *Journal of Behavioral Medicine*.

[11] Tabari M., Ghorbani M. (2009). The Role of Emotional Intelligence in Leadership Decision-making. *Researcher (Management)*.

[12] Mollart L, Skinner V M, Newing C, Foureur M (2013). Factors that may influence midwives work-related stress and burnout. *Women and Birth*.

[13] Farshi Mordaghi Grossi, Arash Mani, Pourroodsari Abbas Bakhshi

[14] Mehdizadeh p, Pourreza A, Allahverdipour H, Dopeykar N (2013). Assessing relationship between job stress, self efficacy and coping among teaching hospitals staff in Tabriz University of Medical sciences in 2009. *Journal of Hospital*.

[15] Görgens Ekermans G, Brand T (2012). Emotional intelligence as a moderator in the stress–burnout relationship: a questionnaire study on nurses. *Journal of clinical nursing*.

[16] Golshiri P., Pourabdian S., Najimi A, Musazadeh H, Hashemi J (2013). factors affecting job stress of nurses working in the emergencies department. *Journal of Health Systems Research*.

[17] Shahabi M, Yamani N., Haghani F

[18] Aramesh f, Badee h (2017). investigating the relationship between spiritual and emotional intelligences and Job stress of auditors. *Journal of Accounting Knowledge, Accounting and Audit Management*.

[19] Goleman D (2012). Emotional intelligence: why it can matter more than IQ.

[20] Goleman D (2013). Working with emotional intelligence.

[21] Anisi J., Majdian M. (2011). Examining the validity of Neo five factors software in students. *Journal of Behavioural Sciences*.

[22] Ebstrup JF, Eplov LF, Pisinger C, Jorgensen T (2011). Association between the Five Factor personality traits and perceived stress: is the effect mediated by general self-efficacy?. *Anxiety Stress Coping*.

[23] Subburaj A, Shunmuga M, Sekar M, Sumathi P (2012). Big five personality traits-a tool for managing stress. *Tactful Management Research Journal*.

[24] Jahanbakhsh Ganjeh S, OmidiArjenaki N, Nori A, Oreyzi HR (2009). The relationship of personality characteristics and burn out among nurses. *Iranian Journal of Nursing and Midwifery Research (IJNMR)*.

[25] Atashrouz B, Pakdaman Sh, Asghari A (2008). The relationship between the big five personality traits and academic achievement. *Journal of Iranian Psychologists*.

